# A Novel 3-D Mineralized Tumor Model to Study Breast Cancer Bone Metastasis

**DOI:** 10.1371/journal.pone.0008849

**Published:** 2010-01-22

**Authors:** Siddharth P. Pathi, Christine Kowalczewski, Ramya Tadipatri, Claudia Fischbach

**Affiliations:** Department of Biomedical Engineering, Cornell University, Ithaca, New York, United States of America; Dresden University of Technology, Germany

## Abstract

**Background:**

Metastatic bone disease is a frequent cause of morbidity in patients with advanced breast cancer, but the role of the bone mineral hydroxyapatite (HA) in this process remains unclear. We have developed a novel mineralized 3-D tumor model and have employed this culture system to systematically investigate the pro-metastatic role of HA under physiologically relevant conditions *in vitro*.

**Methodology/Principal Findings:**

MDA-MB231 breast cancer cells were cultured within non-mineralized or mineralized polymeric scaffolds fabricated by a gas foaming-particulate leaching technique. Tumor cell adhesion, proliferation, and secretion of pro-osteoclastic interleukin-8 (IL-8) was increased in mineralized tumor models as compared to non-mineralized tumor models, and IL-8 secretion was more pronounced for bone-specific MDA-MB231 subpopulations relative to lung-specific breast cancer cells. These differences were pathologically significant as conditioned media collected from mineralized tumor models promoted osteoclastogenesis in an IL-8 dependent manner. Finally, drug testing and signaling studies with transforming growth factor beta (TGFβ) confirmed the clinical relevance of our culture system and revealed that breast cancer cell behavior is broadly affected by HA.

**Conclusions/Significance:**

Our results indicate that HA promotes features associated with the neoplastic and metastatic growth of breast carcinoma cells in bone and that IL-8 may play an important role in this process. The developed mineralized tumor models may help to reveal the underlying cellular and molecular mechanisms that may ultimately enable more efficacious therapy of patients with advanced breast cancer.

## Introduction

Bone metastasis, the spread of tumor cells to the skeleton, is a frequent cause of morbidity and mortality in patients with advanced cancers. Seventy-percent of breast cancer patients with advanced disease develop bone metastases [1], which lead to severe bone pain, pathological fractures, hypercalcaemia, and nerve compression [2]–[4]. These debilitating skeletal events are a consequence of secondary tumor formation and pathological bone remodeling of predominantly osteolytic character [2].

While organ-specific patterns of metastatic colonization are often mediated by site-specific metastasis signatures [5], microenvironmental conditions in the bone are implicated in the pathogenesis of metastatic breast cancer. More specifically, mammary tumor cells localized within bone up-regulate expression of growth factors and cytokines that stimulate osteoclastogenesis and inhibit osteoblast differentiation relative to the same cells located in soft tissue sites [6]–[8]. This process not only promotes pathological bone resorption, but also leads to the release of morphogens that further stimulate metastatic tumor growth and exacerbate the imbalance between bone formation and resorption.

A variety of growth factors and cytokines drive the vicious cycle of bone metastasis by increasing tumor progression and pathological bone remodeling. For example, vascular endothelial growth factor (VEGF) stimulates the formation of new blood vessels and regulates apoptosis and differentiation of tumor cells and osteoblasts [9], [10]. Additionally, parathyroid hormone related protein (PTHrP) and interleukin-11 (IL-11) enhance bone metastasis through activation of osteoclastogenesis [2], [6], [11]–[13] and interleukin-8 (IL-8) may be equally important because of its pro-angiogenic, pro-migratory, and osteoclastogenic activities [7], [14]–[20]. Whether or not specific bone microenvironmental conditions play a role in regulating the signaling by these pro-metastatic factors, however, remains unclear.

The bone extracellular matrix (ECM) is a composite material that consists of an inorganic mineral phase dispersed throughout an organic matrix of collagen. The inorganic component of bone is primarily composed of the mineral hydroxyapatite (HA), a crystalline calcium phosphate phase with the molecular formula Ca_5_(PO_4_)_3_(OH) [21]. While HA is noted for its role in imbuing bone with exceptional tissue stiffness and serving as a reservoir of ions (i.e., Ca^2+^ and PO_4_^3−^), it also represents a bioactive material that modulates the behavior of both normal and transformed cells [21]–[23]. However, the contributions of ECM-associated HA on bone metastasis remain unclear.

Three-dimensional (3-D) polymeric scaffolds represent highly innovative tools for recreating tumor microenvironmental conditions in culture [24]–[27]. Compared to conventional 2-D cultures, tumor cells maintained in 3-D scaffold-based tumor models exhibit characteristics that are more representative of their behavior *in vivo* [25], [26]. We therefore engineered a novel, mineralized 3-D tumor model to investigate the impact of HA on bone cancer metastasis. This system allowed us to examine various stages of secondary tumor growth within bone microenvironments (i.e., initial colonization and subsequent tumor progression, osteolytic capability) under well-defined and pathologically relevant conditions *in vitro*. Our findings indicate for the first time that HA plays a critical role in controlling directing the osteolytic phenotype of breast cancer bone metastasis, underscoring the importance of this mineralized 3-D model system for the study of tumor behavior in bone.

## Results

### Physicochemical Characterization of Scaffolds

**Figure 1:**
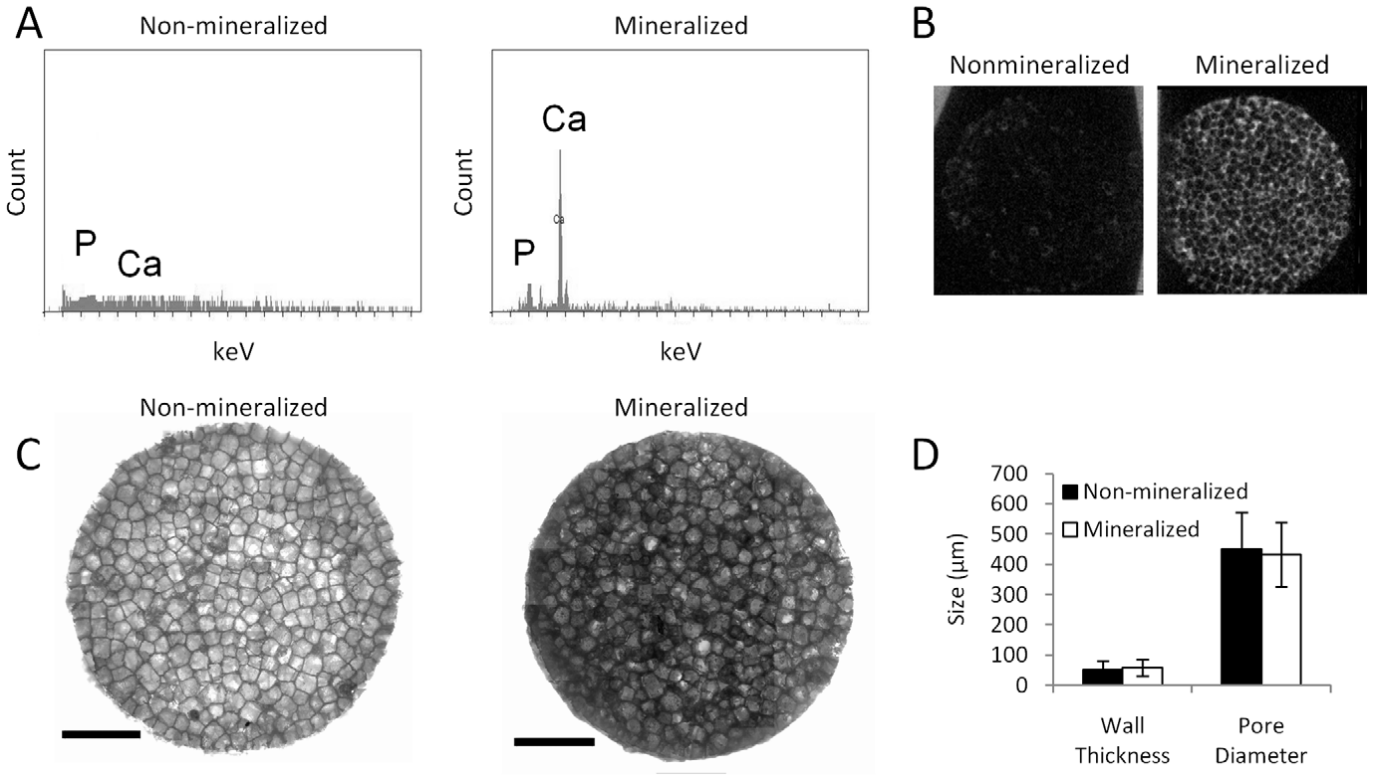
Physicochemical characterization of scaffolds. (A) HA in biomineralized scaffolds is available for cellular interactions as EDS analysis indicates Ca and P at the porous surface of the scaffolds, while no mineral was detected for non-mineralized control scaffolds. (B) MicroCT scans indicate that HA is uniformly distributed throughout biomineralized scaffolds, but was not present non-mineralized control scaffolds. (C) Incorporation of HA did not alter the scaffold microarchitecture relative to non-mineralized control scaffolds as indicated by visualization via brightfield microscopy. Scale bars represent 2 mm. (D) Image analysis of high-resolution brightfield microscopy images indicates that pore size and polymer wall thickness are similar for both biomineralized and non-mineralized scaffolds.

Scaffold characterization was performed to determine the physicochemical properties of the developed matrices. Energy dispersive spectroscopy (EDS) analysis indicated that HA was available for cellular interactions at the porous surface of the mineralized scaffolds, while non-mineralized control scaffolds contained undetectable amounts of calcium or phosphate (Fig. 1A). Additionally, microCT analysis confirmed uniform distribution of HA throughout the mineralized scaffolds, whereas no X-ray absorption was detected for non-mineralized control scaffolds (Fig. 1B). Not surprisingly, mineralized scaffolds exhibited compressive moduli that were 2-fold higher than for non-mineralized control scaffolds (data not shown). These differences were related to the presence of HA as opposed to alterations in the microarchitecture, as mineralized and non-mineralized control scaffolds exhibited similar wall thicknesses (mineralized scaffolds: 58±27 µm, non-mineralized scaffolds: 50±30 µm) and pore diameters (mineralized scaffolds: 432±106 µm, non-mineralized scaffolds: 451±123 µm) (Fig. 1C,D).

### Mineralized Scaffolds Promote MDA-MB231 Adhesion and Proliferation

**Figure 2:**
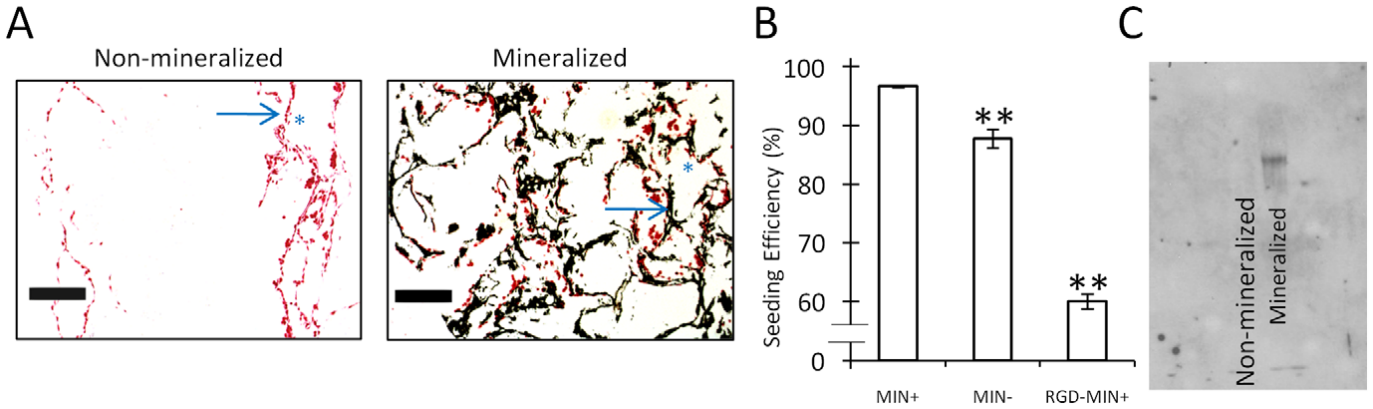
Effect of HA on breast cancer cell adhesion. (A) Analysis of von Kossa stained histological cross-sections indicates that MDA-MB231 cells (stained red) penetrate into the center of mineralized scaffolds (HA stained black), while this is not the case for non-mineralized control scaffolds. Arrows and asterisks indicate representative scaffold walls and pores, respectively. Scale bars represent 200 µm. (B) MDA-MB231 breast cancer cells adhere more efficiently to mineralized scaffolds than non-mineralized scaffolds (**p<0.01), and pre-incubation of MDA-MB231 cells with RGD peptide inhibits enhanced adhesion to mineralized scaffolds (**p<0.01). Error bars are small where not visible. (C) Fibronectin adsorption within the polymer scaffold is increased in mineralized scaffolds (lane 2) relative to non-mineralized scaffolds (lane 1) as indicated by Western Blot analysis of scaffold lysates.

**Figure 3:**
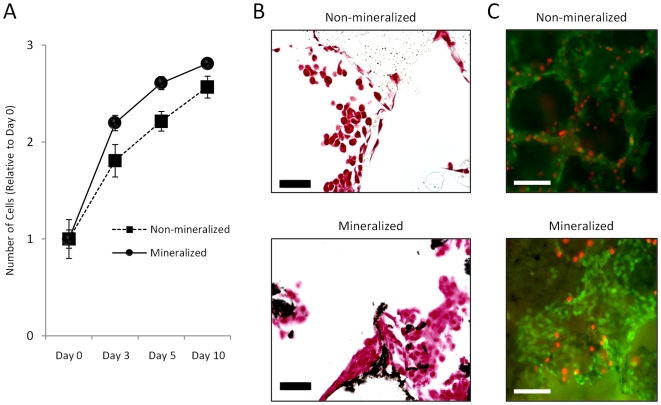
Effect of HA on 3-D tumor tissue formation. (A) Quantification of DNA indicates enhanced proliferation of MDA-MB231 cells within mineralized scaffolds as compared to non-mineralized scaffolds. (B) Analysis of von Kossa stained histological cross-sections revealed that 5 days after seeding, coherent tissue begins to form in mineralized scaffolds but not non-mineralized scaffolds (MDA-MB-231 cells stained red, HA stained black). Scale bars represent 50 µm. (C) Live and dead staining with calcein (green) and propidium iodide (red), respectively, shows increased cell number and tissue formation into pores of mineralized scaffolds relative to control scaffolds. White scale bars represent 100 µm.

The ability of HA to affect breast cancer cell colonization and tumor growth within the bone microenvironment was next assessed. Relative to non-mineralized scaffolds MDA-MB231 cells displayed greater penetration into the center of the mineralized scaffolds and increased adhesion onto HA-containing surfaces, as indicated by von Kossa staining (Fig. 2A). Quantification of seeding efficiency confirmed increased adhesion of tumor cells into mineralized scaffolds (Fig. 2B). These differences were likely related to increased adsorption of adhesion proteins because Western Blot analysis showed that fibronectin was more readily adsorbed onto mineralized scaffolds as compared to non-mineralized scaffolds (Fig. 2C). Furthermore, blockade of fibronectin-binding integrins with soluble arginine-glycine-aspartic acid (RGD) peptides decreased initial adhesion into mineralized scaffolds by 36% (Fig. 2B). Interestingly, the proliferative capacity of MDA-MB231 cells was also increased in the presence of HA, as cell numbers increased in mineralized scaffolds relative to control scaffolds (Fig. 3A). Similarly, MCF-7 cells exhibited a 20% increase in proliferation in mineralized scaffolds (data not shown) verifying the relevance of our findings with a second cell type. In agreement with these results, von Kossa and live/dead staining of the different MDA-MB231 tumor models indicated enhanced tumor tissue formation in the presence of HA after 5 days in culture (Fig. 3B,C).

### Bone Mineral Promotes Tumor-Mediated Osteoclast Differentiation and Activity

**Figure 4:**
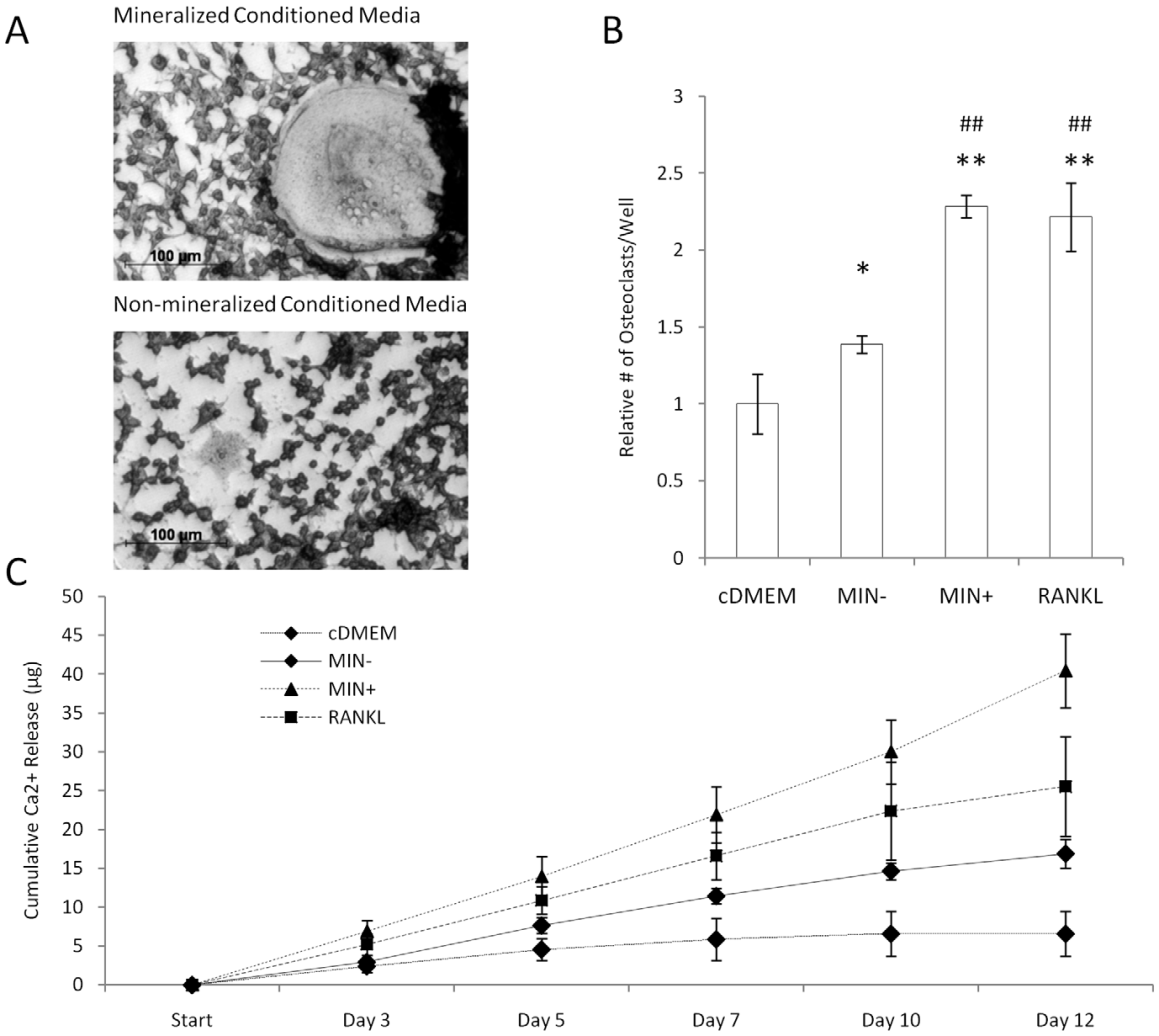
Osteoclastogenesis in response to conditioned media. (A) Conditioned media collected from mineralized tumor models increased RAW 264.7 osteoclastogenesis relative to conditioned media collected from non-mineralized tumor models as revealed by TRAP staining of large multinucleated cells. (B) Quantification of TRAP+ cells indicated that culture media collected from mineralized scaffold cultures (MIN+) promoted RAW 264.7 osteoclastogenesis relative to control media (cDMEM) and media collected from non-mineralized scaffold cultures (MIN-) in a manner that was similar to osteoclastic RANKL. Asterisks [*p<0.05, **p<0.01] and pound signs [#p<0.05, ##p<0.01] indicate statistical significance with respect to ‘cDMEM’ and ‘MIN-’, respectively. (C) Conditioned media collected from mineralized models (MIN+) enhances the resorptive activity of RAW 264.7 relative to all other tested conditions as indicated by 2-D culture on calcium phosphate disks and subsequent analysis of calcium release by a colorimetric assay.

To assess a potential role of HA in the osteolytic phenotype of bone metastases, osteoclastogenesis was evaluated in response to conditioned media. Breast cancer cells cultured within 3-D non-mineralized scaffolds increased osteoclastogenesis as compared to control media that was not exposed to tumor cells and media collected from 2-D cultures (data not shown), but a much more dramatic effect was detected for mineralized tumor models (Fig. 4A,B). More specifically, media from mineralized tumor models increased osteoclast differentiation by 65% with respect to media from non-mineralized scaffold cultures and this effect was comparable to media supplemented with pro-osteoclastic receptor activator for nuclear factor κB ligand (RANKL) (Fig. 4B). To ensure that tumor-derived soluble factors in the mineralized culture media, and not soluble products of the scaffold itself (e.g., Ca^2+^, PO_4_^3−^), were directing osteoclast differentiation, we cultured RAW 264.7 in cell-free scaffold-incubated media and found no differential effects on osteoclastogenesis relative to control media (data not shown). Accordingly, no detectable amounts of Ca^2+^ and PO_4_^3−^ were measured in media collected from non-mineralized or mineralized scaffold cultures. Furthermore, the detected differences in osteoclastogenesis were not due to altered cell proliferation due to similar cell numbers in all tested conditions (data not shown). Evaluation of osteoclast resorptive activity [28] confirmed our results by revealing significantly higher free Ca^2+^ concentrations in response to media collected from mineralized tumor models exceeding even the osteolytic potential mediated by 50 ng/mL RANKL (Fig. 4C).

### Bone Mineral Enhances Tumor-Mediated Osteoclastogenesis through Enhanced Secretion of IL-8

**Figure 5:**
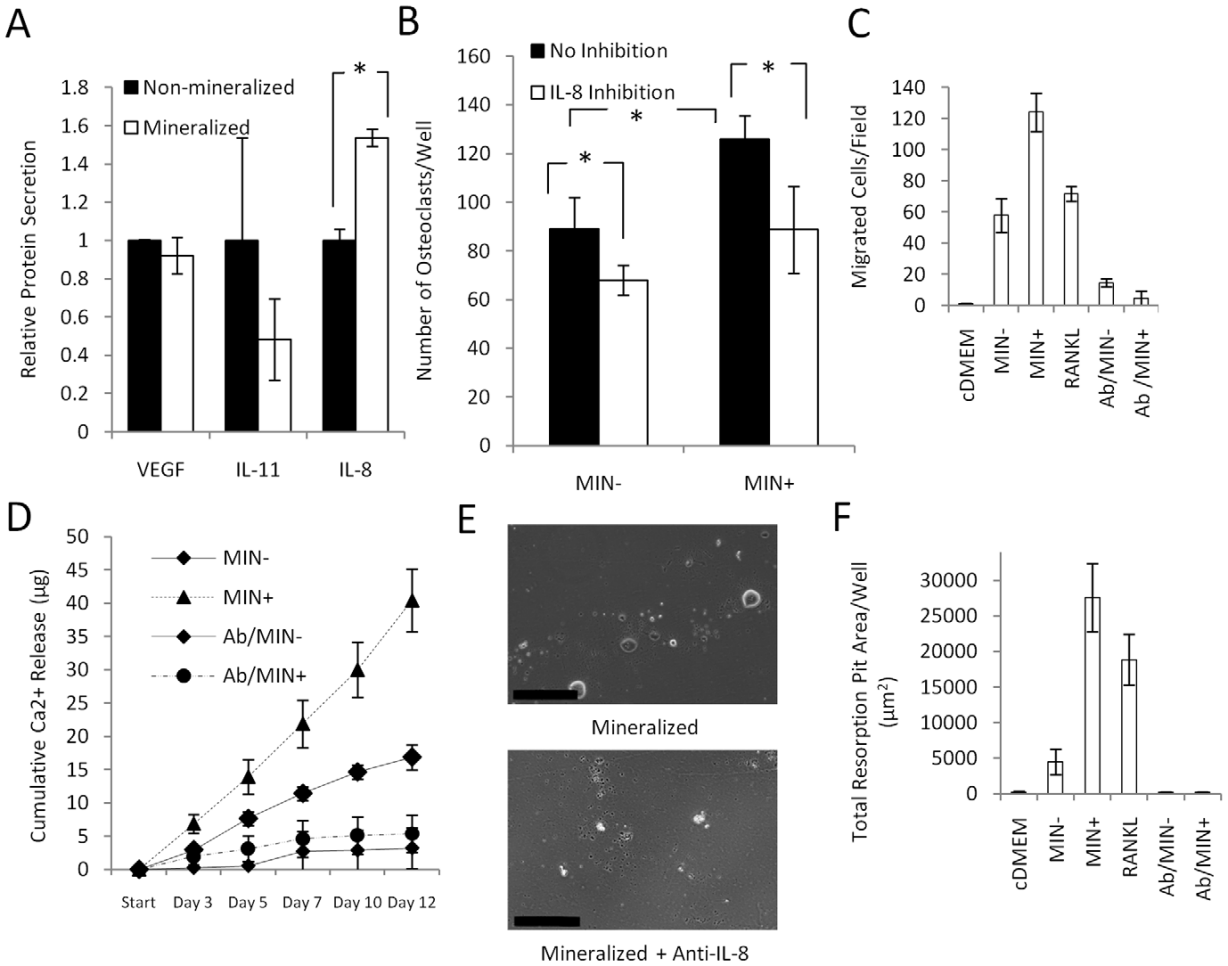
Osteoclastogenesis in response to HA-dependent IL-8 signaling. (A) Tumor cells cultured within mineralized scaffolds up-regulated secretion of IL-8 relative to culture within non-mineralized control scaffolds, while no effect was detected for VEGF and IL-11 secretion (*p<0.05). Error bars for IL-11 are large due to low IL-11 secretion. (B) Blockade of IL-8 signaling by addition of a neutralizing antibody inhibited the pro-osteoclastic effect of conditioned media collected from mineralized tumors (MIN+) to levels comparable to non-mineralized cultures (MIN-) (*p<0.05). (C) Transwell assays with conditioned media indicate that tumor cells cultured within mineralized scaffolds (MIN+) increase the motility of RAW 264.7 relative to all other conditions. Inhibition with a function blocking antibody suggested that this effect was IL-8 dependent. (D) Colorimetric analysis of Ca-release indicates that IL-8 neutralization in media collected from mineralized tumor models (Ab/MIN+) results in a much more pronounced decrease in osteoclast activity as compared to media collected from non-mineralized scaffold cultures (Ab/MIN-). (E) Micrographs of osteoclast-mediated pit formation on bone mineral surface in the presence of conditioned media from mineralized tumor models with and without IL-8 antibody. Scale bars represent 200 µm. (F) Quantification of pit formation in response to the different media in the presence and absence of functional IL-8 signaling.

The ability of HA to modulate the molecular interplay involved in tumor-mediated bone osteolysis was next tested by examining VEGF, PTHrP, IL-11, and IL-8 as specific examples of tumorigenic and pro-osteoclastic factors. While VEGF and IL-11 secretion by MDA-MB231 cells were not affected by the scaffold type (Fig. 5A) and PTHrP was secreted at undetectable levels (<0.3 pM) from either mineralized or non-mineralized tumor models (data not shown), IL-8 secretion was increased by 38% in mineralized scaffolds HA (Fig 5A). Similarly, MCF-7 cells secreted 40% more IL-8 when cultured within mineralized scaffolds as compared to non-mineralized scaffolds (data not shown). These differences were attributed to the inherent bioactivity of HA rather than the different mechanical stiffness of the matrices (non-mineralized scaffolds: 0.5 MPa, mineralized scaffolds: 1.1 MPa), as scaffolds with lower (50% HA) and higher HA content (200% HA) and thus lower (0.7 MPa) and higher elastic modulus (2.1 MPa) exhibited similar effects on breast cancer cell proliferation and IL-8 secretion (data not shown). IL-8 up-regulation mediated pro-osteoclastic effects, because inhibition of this signaling by an IL-8 neutralizing antibody yielded similar numbers of osteoclasts as media collected from non-mineralized scaffolds (Fig. 5B).

Directed migration may be important to the fusion of RAW 264.7 cells to multinucleated, differentiated osteoclasts, and we hypothesized that IL-8 may play a role in this process. Conditioned media collected from mineralized tumor models induced RAW 264.7 migration more significantly relative to media collected from non-mineralized scaffold cultures or media supplemented with 50 ng/mL RANKL. Blockade of IL-8 signaling dramatically reduced motility to levels comparable with cDMEM control media (Fig. 5C). Finally, IL-8 inhibition more dramatically reduced the resorptive activity of RAW 264.7 cells cultured within media from mineralized tumor models relative to media collected from non-mineralized tumor models (Fig. 5D-F).

### Clinical Relevance of 3-D Mineralized Tumor Models

**Figure 6:**
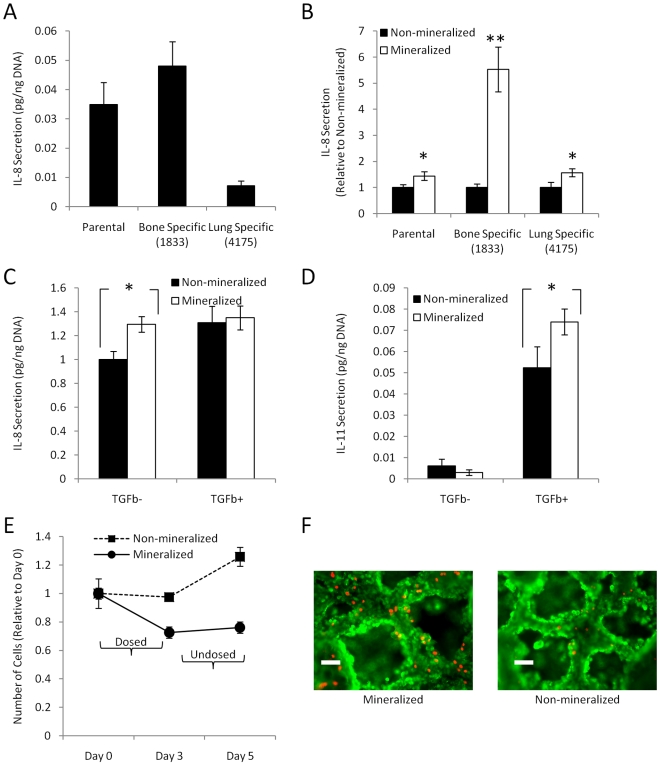
Clinical relevance of 3-D mineralized tumor models. (A) 1833 bone-metastatic MDA-MB231 cells secrete more IL-8 than parental and 4175 lung-metastatic cells in 2-D culture. (B) Mineralized scaffold culture increases IL-8 secretion for all MDA-MB231 populations, but this response is significantly increased in bone-metastatic cells as compared to parental and lung-metastatic cells (**p<0.01, p<0.05). (C) TGFβ1 up-regulates IL-8 secretion in non-mineralized, but not mineralized 3-D cultures of MDA-MB231. (D) In contrast, TGFβ1 increases IL-11 secretion more significantly in mineralized than non-mineralized cultures (p<0.05). (E) Tumor cells cultured within mineralized scaffolds exhibit increased growth suppression in response to ibandronate both when directly exposed to the drug in the dosed interval (days 1–3) and after the drug was removed during the undosed interval (days 3–5). (F) Live/dead staining with calcein (green) and propidium iodide (red) confirmed increased cell death in response to ibandronate in mineralized cultures relative to non-mineralized cultures. Scale bars represent 100 µm.

To verify that HA affects breast cancer cell behavior in a clinically relevant manner we evaluated IL-8 secretion in site-specific metastatic populations of MDA-MB231. Specifically, we utilized bone and lung metastatic breast cells (established by Dr. Massagué [5], [12], [29]–[31]) to assess whether these cells secrete different amounts of IL-8, and if these levels were altered due to HA presence. In conventional 2-D culture, IL-8 secretion was 68% higher in the bone-specific subline (1833) as compared to parental MDA-MB231, while cells of the lung-specific subline (4175), secreted 80% less IL-8 than the parental cell line (Fig. 6A). Interestingly, 1833 cells exhibited a dramatic increase (5.5-fold) in IL-8 secretion in response to HA as compared to parental and lung-specific MDA-MB231 cells (<2-fold) (Fig. 6B) elevating the total concentration of IL-8 in the bone-like microenvironment 3-fold and 6-fold relative to parental and lung-specific MDA-MB231 cells, respectively.

Next, we tested the effect of TGFβ1, an important pro-metastatic factor of the bone metastasis microenvironment, on osteolytic factor secretion. TGFβ1 has been reported to up-regulate IL-8 [32] and we detected increased IL-8 secretion in response to TGFβ1 in non-mineralized cultures, but surprisingly not in mineralized tumor models (Fig. 6C). Interestingly, we noticed a different trend when analyzing the effect of TGFβ1 on IL-11 secretion. While MDA-MB231 cells in non-mineralized scaffolds increased their IL-11 secretion by nearly 10-fold, we detected a greater than 20-fold increase in IL-11 secretion when these same cells were cultured in mineralized scaffolds (Fig. 6D).

As bisphosphonates are the standard of care of bone metastatic breast cancer patients, we also evaluated the therapeutic efficacy of ibandronate in our culture system. Breast cancer cells exhibited increased susceptibility to ibandronate in mineralized scaffolds compared to non-mineralized scaffolds both during administration of the drug (dosed interval), and after the drug was removed from the culture medium (undosed interval) (Fig. 6E, F). The switch between dosed and undosed intervals mimicked clinical treatments in which bisphosphonates are administered intravenously every 3–4 weeks (ZOMETA [package insert]. Stein, Switzerland: Novartis Pharmaceuticals Corp; 2004). These differences were likely associated with binding of ibandronate to HA as tumor cell responsiveness was only detected in mineralized scaffolds pre-incubated with the drug prior to seeding. Importantly, addition of ibandronate to 2-D cultures and pre-cultured 3-D tumor models did not yield changes in tumor cell proliferation or death (data not shown).

## Discussion

By integrating biomaterials and tissue engineering strategies, we have developed a novel, mineralized 3-D tumor model and have employed this culture system to systematically investigate the pro-metastatic role of HA under pathologically relevant conditions *in vitro*. The results of our study indicate for the first time that HA controls critical aspects of breast cancer bone metastasis by directing mammary tumor cells towards a phenotype that promotes secondary tumor growth and bone destruction and regulates drug responsiveness.

Our data suggest that cellular interactions with HA are involved in the colonization and proliferation of breast cancer cells within the bone microenvironment and that RGD-containing fibronectin plays a role in this process. This possible relationship is supported by other studies, which have demonstrated that stromal cell adhesion to HA is mediated by RGD-containing proteins [33], [34] and that a truncated version of fibronectin can block breast cancer growth and metastasis *in vivo* [35]. Accordingly, HA promoted tumor tissue formation within the biomimetic tumor models in our studies and this effect was likely due to the integrated effects of increased adhesion and proliferation. Typically, secondary tumor formation within the bone microenvironment is considered a function of bone resorption because the degrading bone mineral matrix releases bioactive ions and growth factors critical to the proliferation of both normal and transformed cells [4], [11], [36], [37]. Our data suggest that not only soluble, but also insoluble cues inherent to the inorganic component of the bone mineral matrix regulate the metastatic growth of breast cancer cells within bone.

Tumor-derived soluble factors shift the balance between bone formation and bone degradation towards osteolysis [2], [4], [7], [11], [16], [31], [36], and our results indicate that HA is strongly implicated in this process. To identify a potential molecular mediator that may be involved in these effects, we investigated the secretion of a number of growth factors and cytokines that are pivotal to bone metastasis. While VEGF, PTHrP, and IL-11, all of which are critical regulators of tumor angiogenesis [26], [38] and osteoclast resorption [2], [11]–[13], [36], were not affected by the presence of HA, IL-8 secretion increased under these conditions. IL-8 promotes tumorigenesis through inflammatory and pro-angiogenic effects, but increasing evidence additionally implicates IL-8 as a pro-osteoclastic factor [7], [16], [39]. Our data support this concept and additionally suggest that HA increases the pro-osteolytic secretion of IL-8 independent of TGFβ. Essentially our data imply that tumor cells closely associated with the bone matrix may utilize HA-dependent up-regulation of IL-8, while cells located in the center of the tumor (i.e., those not in direct contact with HA) may depend on TGFβ signaling to increase IL-8 secretion. HA may mediate its effects via activation of IL-8 related signaling pathways that may play a role in ruffled-border formation and subsequent bone degradation [40], [41]. Our finding that bone-specific MDA-MB231 cells dramatically increase IL-8 concentrations in the bone microenvironment (both inherently and in response to HA) with respect to parental or lung-specific MDA-MB231 cells provides further evidence of the clinical relevance of IL-8 in regulating breast cancer bone metastasis.

HA may mediate the pro-metastatic effects reported in our studies through a variety of mechanisms. Differences in protein adsorption may support the initial colonization of tumor cells extravasated from blood vessels into the bone microenvironment. Changes in protein adsorption may, in turn, alter the engagement of integrins, which is implicated in IL-8 dependent tumor growth [26]. Differences in cell signaling may therefore not only be related to quantitative differences in protein adsorption as detected in our studies, but also to conformational changes associated with HA-protein interactions [42]. Additionally, the presence of HA may stimulate rises in extracellular Ca^2+^ concentrations, which may affect cancer cell behavior [43]. However, HA is not highly soluble in the absence of strongly acidic conditions, and, accordingly, we did not detect elevated Ca^2+^ concentrations in media collected from mineralized scaffolds. Finally, HA confers the bone ECM with enhanced mechanical stiffness, and physical forces dictate the behavior of normal and transformed cells [44]–[47]. Based on our data, changes in breast cancer cell behavior were influenced by the inherent bioactivity of HA rather than enhanced substrate stiffness. These results do not exclude the possibility that mechanical stiffness influences bone metastasis over a different range of elastic moduli. Not only tissue stiffness, but heightened interstitial pressure (e.g., due to dysfunctional tumor vasculature) may be affecting bone metastasis *in vivo* [45]. Although interstitial pressure was not affected in our tumor models, it will be possible to model these effects in the future to provide an integrated understanding of how the many cues in the microenvironment are synergistically promoting cancer cell colonization and growth.

We have identified that the osteoclastogenic signaling of IL-8 is regulated by HA, but it should be noted that the presence of bone mineral may more broadly affect bone metastasis. For example, HA could alter tumor cell secretion of osteopontin and osteocalcin, two factors that regulate bone cell signaling and have a strong affinity for HA [34], [48]–[51]. Considering the complexity of the bone microenvironment, it will also be important to take into account the contributions of other bone cells such as osteoblasts and osteocytes. These cells release factors that enable tumor cells to promote osteoclastogenesis [2], [6] and this capability may be altered by HA. Specifically, osteoblasts secrete TGFβ1 in a manner that depends on the specific bone characteristics [52], and that may promote bone resorption via increasing IL-11 secretion [11]–[13], [31], [53], [54]. Our findings now additionally suggest that cancer cell response to TGFβ1 is regulated by material interactions with HA. Future studies, integrating HA with 3-D co-culture of multiple bone cell types will help to further reveal the underlying signaling.

Bisphosphonates inhibit osteoclast activation by binding to mineralized surfaces [2], [36], and in our culture system, tumor cell response to ibandronate was increased in the presence of HA. These results agree with previous *in vivo* studies in which ibandronate treatment decreased MDA-MB231 tumor burden at the bony site, but not in the mammary fat pad [55]. Our observation that ibandronate halts tumor cell proliferation even after removal of the drug from the culture medium could further explain why breast cancer patients treated with bisphosphonates have increased disease-free survival and a significantly reduced risk of disease progression [56]. We saw no effect of ibandronate on the breast cancer cells in 2-D or in pre-cultured 3-D models with existing tissue formation, supporting our contention that drug efficacy is due to the effects of HA binding. Collectively, our data suggest that bisphosphonates bind to and integrate with mineralized surfaces to deter disease progression and cellular growth at the bony site.

In conclusion, HA induces secondary tumor growth and IL-8 secretion by breast cancer cells, providing a molecular mechanism by which the mineral matrix of the bone microenvironment regulates the pathological bone remodeling associated with breast cancer bone metastasis. Directly targeting HA as an integral component of the bone mineral matrix may not represent a viable option for therapy. However, HA functions may be explored for improved treatments with bisphosphonates. Tissue-engineered tumor models will enable studies that will lead to an improved understanding of the molecular mechanisms by which HA regulates bone metastasis and current treatment strategies. This information will be critical to the identification of novel and specific therapeutic targets that will permit the improved therapy of patients with advanced breast cancer.

## Materials and Methods

### Cell Culture

Human MDA-MB231 [5], [26], [30], [36] (ATCC), bone-metastatic (1833) and lung-metastatic (4175) MDA-MB231 subpopulations [30], [31] (kindly provided by Dr. Joan Massague), MCF-7 [8], [25], [36] (ATCC), and murine monocytic RAW 264.7 [57]–[59] cells (ATCC) were routinely maintained in complete DMEM (cDMEM) (i.e., DMEM (Invitrogen) supplemented with 10% fetal bovine serum [FBS, from Tissue Culture Biologicals] and 1% penicillin/streptomycin [PS, from Invitrogen]), under standard cell culture conditions (37°C, 5% CO_2_).

### Scaffold Fabrication

Porous mineralized scaffolds, composed of poly(lactide-co-glycolide) (PLG) and hydroxyapatite (HA), were fabricated by a modified gas forming/particulate leaching method [60]. Briefly, scaffolds were prepared with PLG particles (Lakeshore Biomaterials; average diameter = 250 µm), PLG microspheres (formed through a double emulsion process, average diameter = 5–50 µm), HA particles (Sigma, average diameter of 200 nm confirmed with transmission electron microscopy), and sodium chloride particles (sieved to a diameter of 250–400 µm, J.T. Baker). A total of 8 mg of polymer, 8 mg of HA nanoparticles, and 152 mg of NaCl was used and resulted in final scaffold dimensions of 8.5 mm diameter by 1 mm thickness after cold-pressing with a Carver Press (Fred S. Carver). Following high-pressure immersion in carbon dioxide gas and polymer foaming in a non-stirred pressure vessel (Parr Instruments), the scaffolds were leached in de-ionized water for 24 hours to remove NaCl porogen particles. Non-mineralized PLG scaffolds, which were HA-free, were also fabricated, as were mineralized scaffolds with varying amounts of HA (1∶2, 1∶4, 2∶1 mass ratio with PLG). Prior to cell culture, scaffolds were sterilized by submersion in 70% ethanol for 30 minutes followed by 5 washes in sterile PBS.

### Scaffold Characterization

Scanning electron microscopy (SEM) (Leica 440) and light microscopy (Observer.Z1, Zeiss) were used to characterize the microarchitecture and porosity of scaffolds. Energy dispersive spectroscopy (EDS) (Leica 440) was employed to provide elemental analysis of scaffolds, including determination of surface presentation of HA in mineralized scaffolds. For SEM and EDS, scaffolds were sputter-coated with gold-palladium to reduce surface charge buildup (Denton Desk II). Scaffolds were tested with MicroCT (GE Healthcare, eXplore CT120) for further microarchitectural analysis and to ensure uniform distribution of HA in mineralized scaffolds. Dynamic mechanical analysis (TA Instruments Q800) was used to determine the compressive moduli of the scaffolds.

### Western Blot Analysis

Unseeded scaffolds were incubated in cDMEM for 30 minutes, allowing serum proteins to adsorb. Subsequently, the adsorbed proteins were released from the scaffolds by mechanically disintegrating the polymer matrices in a solution of RIPA buffer (Sigma), protease inhibitor cocktail (Sigma), and PMSF (Sigma). Following centrifugation, the resulting scaffold lysates were resolved by SDS-PAGE and were blotted onto a PVDF membrane (BioRad). The membrane was incubated overnight with a rabbit anti-human fibronectin (Sigma) antibody. The membrane was then washed and incubated with a species-specific HRP-conjugated secondary antibody (Novus Biologicals), followed by ECL detection (Amersham Biosciences).

### Development and Characterization of 3-D Mineralized Tumor Models

1.5 million breast cancer cells suspended in 30 µL cDMEM were seeded into each scaffold and maintained under dynamic culture conditions on an orbital shaker at 37°C and 5% CO_2_ for up to 10 days. In some cases, the culture medium was supplemented with 200 pM of recombinant human TGFβ1 (R&D) or 1 µM ibandronate (Sigma) based on dose dependency studies [12], [29], [55]. Live/Dead assay was performed with calcein/propidium iodide (Invitrogen) staining and visualization on an Epi-fluorescence microscope (Observer.Z1, Zeiss). Cell adhesion was determined by quantifying the number of non-adhered cells 30 min after scaffold seeding using a cell counter (Beckman-Coulter). Blockade of RGD-binding integrins was performed by incubating the cells with 20 µg/mL of soluble RGD (Sigma) for 30 min prior to seeding. To measure cell proliferation, tumor cell-seeded constructs were washed in PBS and lysed in Caron's buffer by sonication. Following centrifugation the DNA content in the supernatant was quantified through fluorescent Hoechst Assay (Invitrogen). For histological analysis tumor constructs were fixed in 10% formaldehyde and embedded in paraffin for sectioning and staining (H&E, von Kossa) according to established protocols.

### Analysis of Conditioned Media

Prior to each time point, breast cancer cell seeded-scaffolds were transferred to fresh culture plates, and media was changed to DMEM/1% FBS. Conditioned media were harvested after 24 hours for analysis. VEGF, IL-11, and IL-8 ELISAs (R&D) were performed on these samples according to manufacturer's instructions, while PTHrP secretion was measured using a two-site immunoradiometric assay (Beckman Coulter). Protein secretion was normalized to DNA content as determined by fluorimetric Hoechst Assay. Scaffold-conditioned control media (i.e. media incubated with unseeded scaffolds) was also analyzed for calcium and phosphate ion concentration by atomic absorption.

### Osteoclastogenic Response

#### TRAP stain

RAW 264.7 monocytes were seeded in tissue culture plates and cultured in tumor-conditioned media collected from mineralized and non-mineralized 3-D tumor models for 5 days with or without 10 µg/mL of IL-8 neutralizing antibody (R&D). cDMEM and scaffold-conditioned control media (i.e. media incubated with unseeded scaffolds) were used as a negative controls, while cDMEM supplemented with RANKL (Sigma) served as a positive control [61]. RANKL was added at a concentration of 50 ng/mL, which has previously been shown to induce osteoclastogenesis [7]. To quantify osteoclastogenesis, a tartrate-resistant acid phosphatase (TRAP) staining kit (Sigma) was used according to manufacturer's instructions in conjunction with image analysis of TRAP positive, multinucleated cells using AxioVision software (Zeiss). Similar cell numbers between the different experimental conditions were verified with an AlamarBlue assay (AbD Serotec Ltd) as indicated by the manufacturer.

#### Migration assay

RAW 264.7 monocytes were seeded on top of collagen-coated polycarbonate tissue culture inserts (8 µm pore diameter, Nunc), which were placed into well plates with tumor-conditioned media or control media as specified above. After 8 h of incubation, a cotton swab was used to remove cells from the top of the inserts, while cells that had migrated through the polycarbonate membrane towards the conditioned media, were stained with DAPI. Stained inserts were then imaged, and the number of migrated cells was analyzed by AxioVision (Zeiss) image analysis.

#### Osteoclast activity assays

RAW 264.7 monocytes were seeded onto Corning Bone Cell Assay Surfaces [28] (kindly provided by Corning Life Sciences) and cultured in the culture media specified above. Osteoclast activity was determined by quantifying calcium release from the Bone Cell Assay Surfaces into culture media due to osteoclast-mediated resorption. Specifically, calcium release was measured by colorimetric detection of complexation of free calcium ions to o-cresolphthalein complexone (Sigma). Additionally, osteoclast activity was determined by light microscopic quantification of resorption pit area on the Bone Cell Assay Surface plates.

### Statistical Analysis

One-way ANOVA and Student's t-test were used to determine statistical significance, and p<0.05 is indicated by (*) while p<0.01 is indicated by (**) in all figures. For all experiments, sample size is greater than or equal to 3 for each condition. Data are presented as average; error bars indicate standard deviation.
